# The initiation of nocturnal dormancy in *Synechococcus* as an active process

**DOI:** 10.1186/s12915-015-0144-2

**Published:** 2015-06-10

**Authors:** Sotaro Takano, Jun Tomita, Kintake Sonoike, Hideo Iwasaki

**Affiliations:** Department of Electrical Engineering and Biological Science, Waseda University, TWIns, Shinjuku, Tokyo 162-8480 Japan; Department of Neuropharmacology, Graduate School of Pharmaceutical Sciences, Nagoya City University, Mizuhoku, Nagoya 467-8603 Japan; Faculty of Education and Integrated Arts and Sciences, Waseda University, TWIns, Shinjuku, Tokyo 162-8480 Japan

**Keywords:** Cyanobacteria, Light/Dark, Transcription, Feed-forward regulation

## Abstract

**Background:**

Most organisms, especially photoautotrophs, alter their behaviours in response to day–night alternations adaptively because of their great reliance on light. Upon light-to-dark transition, dramatic and universal decreases in transcription level of the majority of the genes in the genome of the unicellular cyanobacterium, *Synechococcus elongatus* PCC 7942 are observed. Because *Synechococcus* is an obligate photoautotroph, it has been generally assumed that repression of the transcription in the dark (dark repression) would be caused by a nocturnal decrease in photosynthetic activities through the reduced availability of energy (e.g. adenosine triphosphate (ATP)) needed for mRNA synthesis.

**Results:**

However, against this general assumption, we obtained evidence that the rapid and dynamic dark repression is an active process. Although the addition of photosynthesis inhibitors to cells exposed to light mimicked transcription profiles in the dark, it did not significantly affect the cellular level of ATP. By contrast, when ATP levels were decreased by the inhibition of both photosynthesis and respiration, the transcriptional repression was attenuated through inhibition of RNA degradation. This observation indicates that *Synechococcus* actively downregulates genome-wide transcription in the dark. Even though the level of total mRNA dramatically decreased in the dark, *Synechococcus* cells were still viable, and they do not need *de novo* transcription for their survival in the dark for at least 48 hours.

**Conclusions:**

Dark repression appears to enable cells to enter into nocturnal dormancy as a feed-forward process, which would be advantageous for their survival under periodic nocturnal conditions.

**Electronic supplementary material:**

The online version of this article (doi:10.1186/s12915-015-0144-2) contains supplementary material, which is available to authorized users.

## Background

Most organisms, ranging from bacteria to higher plants and animals, change their behaviour in response to day–night alternations. In particular, photosynthetic organisms alter their intracellular activities adaptively because of their great reliance on light. The unicellular cyanobacterium, *Synechococcus elongatus* PCC 7942 (*Synechococcus*, hereafter) is distinguished by its extraordinary change in global transcriptional profile dependent on light or darkness. When *Synechococcus* cells are transferred from the light to the dark, transcription of most of the genes on the genome is dramatically and rapidly suppressed (dark repression), while the transcription of a small subset (about 5 %) of genes is up-regulated (dark induction). Consequently, there is a dramatic reduction in the total transcript level, reaching about 20 % within 12 hours [1], while its mechanism is yet to be elucidated fully. We note that the circadian clock is not essential for the dark-dependent genome-wide transcriptional change, while it modulates some dark-/light-induced transcription in a time-of-day-dependent manner [1, 2].

Because *Synechococcus* is an obligate photoautotroph, we initially expected that dark acclimation would affect the intracellular environment greatly through inhibition of photosynthesis, possibly accompanying reduction of the photophosphorylation-derived ATP level. Doolittle suggested a plausible decrease in the rate of energy-consuming RNA synthesis upon partial inhibition of photosynthetic activity [3]. Accordingly, some studies reported that intracellular ATP content decreases within several hours by the dark incubation in *Synechococcus* [4, 5]. Thus, inhibition of photosynthesis would repress genome-wide transcription generally, by primarily inhibiting energy-requiring mRNA synthesis.

In the present study, we found that treatment with two photosynthesis inhibitors under illumination mimicked nocturnal transcriptional suppression, and the level of photosynthesis inhibition under illumination was actually correlated with that of transcriptional repression. This observation supports the hypothesis that nocturnal depression of the transcription occurs through the cessation of photosynthesis as we expected. However, dark incubation or inhibition of photosynthesis did not reduce cellular ATP content significantly even after transcriptional repression started, at least under our experimental conditions. Therefore, shortage of cellular ATP content would not be a main cause of repressing ATP-requiring transcription in the dark. Conversely, when cellular ATP content in the dark was reduced by inhibiting respiratory electron flow, the dark-induced transcriptional repression was attenuated, thereby keeping the total mRNA at a higher level compared with that under dark conditions. These findings strongly suggest that nocturnal transcriptional suppression is an active process requiring ATP mainly for degrading mRNA.

*Synechococcus* cells under illumination require *de novo* transcription for their survival, while we found that the dark-acclimated cells were able to survive for at least 48 hours without *de novo* mRNA synthesis, as if they became dormant in the dark. These observations suggest that the nocturnal transcriptional attenuation is a predictive feed-forward regulation before the cells experience the critical ATP reduction following longer (approximately 12 hours) dark incubation.

## Results and discussion

### Genome-wide transcriptional repression triggered by inhibition of photosynthetic activity

**Fig. 1:**
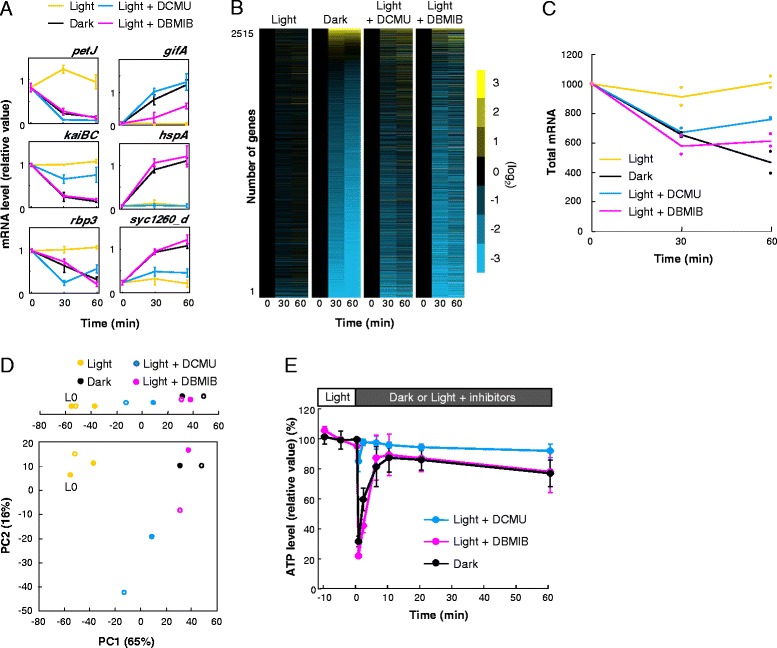
Inhibition of photosynthetic electron transport mimicked dark-repression-like genome-wide transcription profiles under illumination without dramatic loss of ATP content. **a** Temporal expression profiles of representative dark-repressed or -induced genes using three independent northern hybridisation analyses. We normalized the data for dark-repressed genes to the average value of illuminated samples (0, 30, and 60 minutes), while the data for dark-induced genes were normalized to the average value of dark-incubated samples (30 and 60 minutes). Bars indicate the standard deviation. **b** Organisation of *Synechococcus* expression profiles in the light, dark, and light with two inhibitors, DCMU and DBMIB. The data of all genes were normalized to the value at time 0 (minutes), corresponding to 12 hours in the light, and sorted by induction levels in the dark. **c** Total mRNA pools estimated from the sum of mRNA hybridisation signals normalized to genomic DNA signals under each condition. We normalized the signals at time 0 in the light to 1,000. Plots indicate the results from each independent experiment (n = 2). **d** The plot of PCA scores. Upper plot shows the PC1 score of each profile only. Filled circles and open circles indicate the samples at 30- and 60-minutes incubation, respectively. L 0 indicates scores of samples at time 0. For panels B to D, we used averaged data from two independent experiments. **e** Transition of the ATP level when photosynthetic activity was inhibited partially or completely. We transferred cells grown in the light for 12 hours to each condition at time 0. We normalized the ATP levels to the average value of control samples collected in the light at Time −10 to 0 (minutes). Bars indicate the standard deviation from triplicate cultures *DBMIB* 2,5-dibromo-3-methyl-6-isopropylbenzoquinone, *DCMU* 3-(3,4-dichlorophenyl)-1,1-dimethylurea, *PCA* principal component analysis

To examine whether inhibition of photosynthesis triggers dark repression/induction even under light, we applied two photosynthesis electron transport inhibitors, 3-(3,4-dichlorophenyl)-1,1-dimethylurea (DCMU) and 2,5-dibromo-3-methyl-6-isopropylbenzoquinone (DBMIB) (for target sites, see Additional file 1: Figure S1A), and analysed the effects on dark-repressed/induced gene expression profiles. Note that we have confirmed that either 2 μM DCMU or 10 μM DBMIB was sufficient to block electron transport completely by monitoring the effective quantum yield of Photosystem II (Φ_II_), (Fm′–Fo)/Fm′ becoming approximately equal to zero in the light (see Additional file 1: Figure S1B). Cells were grown in the light, acclimated to two 12 hour/12 hour light–dark (LD) cycles, and then returned to the light. At 12 hours in the light after the LD cycles, we kept cells in the light, acclimated them to the dark, or treated them with each of the inhibitors under the light for 30 or 60 minutes, and then subjected the cells to transcription analyses. We observed the changes after 30 minutes and 60 minutes from the addition of the stimuli because the rate of transcript variability is highest in 30 minute dark incubation, and total mRNA decreased up to about 50 % within 60 minutes [2]. Initially, we performed northern blot analyses on three representative dark-repressed genes (for information on genes, see Additional file 2: Table S1), which were remarkably repressed and induced upon light-to-dark transition within 30 minutes, respectively (Fig. 1a). In the cells treated with DCMU or DBMIB under illuminated conditions, the levels of expression of dark-repressed genes, *petJ*, *kaiBC*, and *rbp3*, decreased, while those of dark-induced genes, *gifA*, *syc1260_c*, and *hspA*, increased (Fig. 1a). As exemplified by *hspA*, which was less up-regulated by DCMU than by DBMIB, the extent of dark repression/induction seemed generally greater with DBMIB than with DCMU. These data suggest changes in the expression of all six genes through the cessation of photosynthesis in the dark. Note that longer treatments with DCMU or DBMIB up to eight hours also down-regulated and up-regulated the expression of *kaiBC* and *gifA* under illumination (Additional file 3: Figure S2). Although the level of expression of *kaiBC* was not reduced to zero, even without DBMIB it is reduced within eight hours because of its circadian clock function [6]. Moreover, treatment with DBMIB [7, 8] and DCMU [9] affects circadian clock function, possibly through antagonising the function of KaiA by enhancing the phosphorylation of KaiC. Therefore, longer treatment with these inhibitors may cause complex effects on transcriptional profiles of clock-controlled gene expression, as exemplified by *kaiBC* and many other representative dark-repressed genes. To avoid this confusion, we further focused on the transcriptional and metabolic changes within one hour after dark-acclimation or inhibitor treatment, which should be sufficient to dissect the mechanism triggering dark-induced global expression changes.

Subsequently, we performed a DNA microarray analysis to examine global gene expression profiles in the presence or absence of the inhibitors. As shown in Fig. 1b and c, our DNA microarray analysis confirmed that the levels of most transcripts decreased immediately after dark acclimation so that the total mRNA levels decreased to about 50 % of those in the light within 60 minutes, while a subset of genes, shown in yellow in Fig. 1b, were up-regulated, as reported previously [1, 2]. The expression of most genes in the presence of either DCMU or DBMIB under illuminated conditions was similar to that in the absence of inhibitors under dark conditions (Fig. 1b). The microarray profiles of any of the above-mentioned six representative genes (see Additional file 4: Figure S3A) were essentially the same as those observed by northern analysis (Fig. 1a), validating the method. As shown in Fig. 1c, treatment with DCMU or DBMIB in the light reduces total mRNA levels, as does dark acclimation, while the magnitude of the reduction was somewhat larger with DBMIB than that with DCMU. To compare these transcription profiles more comprehensively, we performed a principal component analysis (PCA). Figure 1d shows the results of the PCA and indicates that the difference in transcription profiles between the illuminated samples and dark-acclimated samples is represented primarily in PC1. Moreover, PC1 and PC2 account for 67 % and 13 % of the variance of expression data, respectively, such that PC1 accounts for 2,515 variables more than PC2. To discuss how closely inhibition of photosynthesis under illumination imitated the transcription profiles of the samples incubated in the dark, we analysed the effects of the inhibitors using the PC1 scores. The top of Fig. 1d shows the projection onto the first principal component. In the presence of DCMU or DBMIB, the plots of PC1 scores were closer to those of dark-acclimated samples than those of illuminated samples. In addition, the PC1 scores of DBMIB-treated samples were closer to those of dark-incubated samples than to those of DCMU-treated samples. Scatter plot analyses were performed to confirm these results by comparing the induction ratio of all tested genes upon dark transition, and the results from the addition of the inhibitors under continuous illumination (see Additional file 5: Figure S4A). Again, treatment with two inhibitors under illuminated conditions caused similar expression changes to dark incubation, while DBMIB resulted in effects more similar to effects seen in dark acclimation as observed in the scatter plots and correlation value than effects seen after DCMU treatment.

We also investigated which genes were significantly expressed in continuously illuminated samples under each condition. We used a modified version of Dunnett’s *t* test (Mulcom test [10]), which has been used in our previous microarray analysis [2]. By using this test, we initially identified 1,844 genes that were significantly dark-regulated (either dark-repressed or -induced), encompassing about 70 % of all tested (2,515) genes. Among the dark-regulated genes, 1,618 (about 88 %) significantly changed their expression patterns after treatment with DCMU or DBMIB, strongly supporting that most of the dark-regulated transcriptional changes are a result of inhibition of photosynthesis (see Additional file 6: Figure S5A). In addition, the number of the genes with altered expression levels after treatment with DBMIB is larger than that after treatment with DCMU (see Additional file 6: Figure S5A), as consistent with the previously mentioned tendency. That is probably because DCMU only blocks the linear electron flow, while DBMIB inhibits both linear and cyclic electron flows (see Additional file 1: Figure S1A). Thus, treatment with DBMIB inhibits photosynthesis more stringently (i.e., more closely mimics the dark condition) than DCMU, such that the levels of cellular activity requiring photosynthesis (e.g. photophosphorylation) are correlated with that of transcriptional changes.

### Suppression of genome-wide transcription without reduction of ATP

**Fig. 2:**
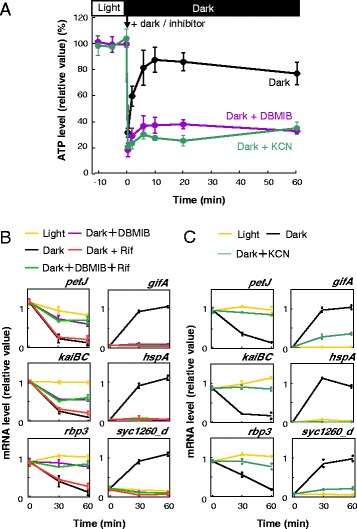
Requirement of ATP maintenance for transcription in representative dark repressed or induced genes under dark conditions. **a** Loss of ATP recovery in the dark resulting from the respiratory electron transport inhibitor, DBMIB or the inhibitor of cytochrome c oxidase, KCN. We analysed and represented data as shown similarly in Fig. 1e. **b** Temporal expression profiles of representative dark repressed or induced genes obtained from three independent northern hybridisation analyses when DBMIB was used as the inhibitor of respiration. **c** Attenuated dark-induced transcriptional changes for representative dark repressed or induced genes when the cells were treated with KCN for the inhibition of respiration. Each plot shows the results of two independent northern blot analyses. For panels b and c, data representation is consistent with that in Fig. 1a *DBMIB* 2,5-dibromo-3-methyl-6-isopropylbenzoquinone

Among various intracellular changes upon dark acclimation, dramatic reduction of ATP could be a major trigger of dark-induced transcriptional repression. The stronger suppressive effect of DBMIB on mRNA level than that of DCMU is also consistent with this possibility, because the inhibition of both linear and cyclic electron flows with DBMIB would result in greater effects on ATP synthesis than inhibiting linear electron flow with DCMU alone (see Additional file 1: Figure S1A). Rust *et al*. [5] reported that the ratio ATP/(ATP + ADP) was reduced within several hours in the dark after transfer from light; however, it did not change much after incubation in the dark for one hour, while the same group more recently reported that it was reduced up to about 60–70 % of that in the light within one hour in the dark under a different illumination schedule with lower light intensity [11]. Therefore, we checked the change of ATP content under our experimental conditions upon light-to-dark transition in more detail using an ATP-luciferase assay. After cells were transferred to the dark, their ATP level transiently decreased, reaching about 30 % of that under illumination, while it rapidly recovered up to 80–90 % within 10 minutes. This level was maintained for at least for one hour (Figs. 1e and 2a, black lines). Note that maintaining about 80–90 % of the ATP content at one hour after dark acclimation is consistent with findings by Rust *et al*. [5]. We interpreted this recovery as the energy supply from respiratory electron transfer in the dark because some studies of ATP synthesis in cyanobacteria indicated that ATP synthesis in the dark must primarily rely on respiration (e.g. [12–16]). Consistent with this assumption, addition of DBMIB or KCN, inhibitors of respiratory electron transport [16], inhibits the recovery of ATP levels in the dark (Fig. 2a, purple and green lines).

Surprisingly, however, under light conditions we observed the recovery of ATP levels after addition of DBMIB, which should inhibit photosynthesis as well as respiration (Fig. 1e, magenta line). By contrast, under light conditions the ATP levels dramatically decreased upon addition of DCCD, an inhibitor of F1Fo-ATPase (see Additional file 7: Figure S6). Taken together, the results suggest the formation of ATP involving a proton gradient formed by the light-driven electron transfer that would detour re-oxidation of plastoquinone at the cytochrome *b*_6_/*f* complex. Alternative oxidases that directly oxidise plastoquinol by oxygen would be candidates because of the electron detour route. Indeed, the presence of a DBMIB- or cyanide-insensitive respiratory pathway has been reported [16, 17]. However, these alternative electron pathways are not generally assumed to contribute to ATP production. Moreover, the involvement of either Photosystem (PS) I or PS II is rather difficult to assume. Because the electron transfer rate through PS II estimated from the parameter Φ_II_, decreased to about 3 % of basal levels upon addition of DBMIB (Additional file 1: Figure S1B), the involvement of PS II in the process of ATP formation should be limited. Similarly, involvement of PS I would be difficult to assume, judging from the fast oxidation kinetics of P700 upon illumination in the presence of DBMIB (Additional file 8: Figure S7). Apparently, DBMIB is able to diminish the electron transfer to P700. A plausible explanation for the formation of ATP in the presence of DBMIB would be the contribution of the previously mentioned remaining 3 % electron transfer in photosynthesis. Because the maximum rate of photosynthetic electron transfer is generally 10 times higher than that of respiratory electron transfer in cyanobacteria (e.g. [18]), the 3 % electron transfer in photosynthesis would correspond to 30 % of the electron transfer in respiration. This 30 % activity may be sufficient for the maintenance of the cellular ATP level.

These observations seem to contradict the hypothesis that shortage of ATP induces a dark-triggered dynamic shift in transcription. Although a transient reduction in the ATP content could be involved in triggering some transcriptional shift upon light-to-dark transition, we suggest this effect is not essential. It is primarily because the dark-stimulated transcriptional shift is evident even after rapid recovery of the ATP concentration (in the light with DBMIB or in the dark). Moreover addition of DCMU induced at least in part of dark-mimicking genome-wide transcriptional change (Fig. 1a-d), while it barely affected ATP content in the light (Fig. 1e). Doolittle predicted that DCMU in the light would reduce the energy source and limit energy available for mRNA synthesis [3], but this is not consistent with our findings.

Generally, in our experimental conditions under illumination, DBMIB showed strong effects mimicking dark-induced transcriptional control. Adding the two photosynthesis inhibitors to *Synechococcus* is assumed to inhibit electron transport to PS I, with DBMIB having a stronger effect than DCMU [19]. To explore the redox state of the components on the acceptor side of PS I under our experimental conditions, we examined oxidation levels and re-reduction kinetics of P700, the reaction centre of PS I, by absorbance changes (Δ*A*_810_; Additional file 8: Figure S7). Just after starting the irradiation, the absorbance level increased to plateau at moderate levels. Addition of either DCMU or DBMIB increased the absorbance level constitutively at higher levels. These results indicate that addition of these two inhibitors oxidises the components downstream of PS I in a manner similar to that during dark incubation. The rates of electron input to PS I were quantified from the half-decay-time of re-reduction of P700 upon post-illumination. Treatment with DCMU or DBMIB resulted in slower reduction kinetics, more so with DBMIB than with DCMU. Thus, it is plausible that the redox state downstream of PS I is important for triggering the dark-induced transcriptional depression.

### Requirement of ATP accumulation for dark-induced transcriptional depression

We further investigated how forced reduction of ATP levels affects the genome-wide transcriptional profile in the dark. We attempted to block the respiratory electron flow, which is shared with photosynthetic electron flow in cyanobacteria (see Additional file 1: Figure S1A), with DBMIB in the dark. As is mentioned above, treatment with DBMIB inhibited the recovery of ATP levels in the dark, as did that with KCN (Fig. 2a). Surprisingly, dark-induced transcriptional suppression and activation were both significantly attenuated by the addition of DBMIB (Fig. 2b). In the presence of DBMIB, for example, dark-induced transcriptional suppression of *petJ*, *kaiBC*, and *rbp3* was largely attenuated (Fig. 2b) while dark-induced up-regulation of *gifA*, *hspA*, and *syc1260_d* was arrested (Fig. 2b). We also confirmed that the dark-induced repression and activation of the six representative genes were similarly attenuated in the presence of KCN in the dark, as they were in the presence of DBMIB (Fig. 2c).

**Fig. 3:**
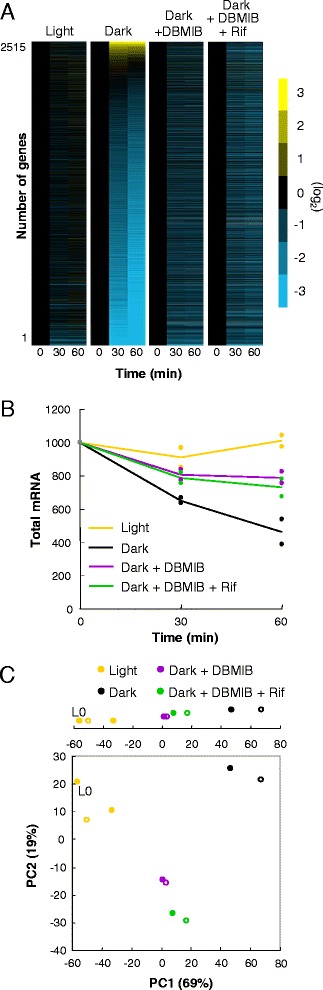
Genome-widely attenuated dark repression and induction by inhibition of ATP synthesis. **a** Organisation of expression profiles in the light, in the dark without inhibitor, or in the dark treated with DBMIB alone or both DBMIB and rifampicin. Data representation is consistent with that in Fig. 1b. **b** Total mRNA accumulation levels at each condition. Data representation is consistent with that in Fig. 1c. **c** Plot of PCA scores. Data representation is consistent with that in Fig. 1d. We obtained the data for panels a to c from independent duplicate experiments *DBMIB* 2,5-dibromo-3-methyl-6-isopropylbenzoquinone, *PCA* principal component analysis

Figure 3a–c shows consistent attenuation of the dark-dependent expression profile of the entire genome. mRNA abundance was reduced to about 50 % upon the dark transition, while in the presence of DBMIB about 80 % of mRNA remained (Fig. 3b). Therefore, DBMIB treatment inhibited about 60 % of dark-dependent mRNA suppression. Figure 3c shows PCA expression profiles, in which PC1 and PC2 account for 69 % and 19 % of the variance of expression data, respectively. As is shown in Fig. 1d, the difference between the illuminated and dark-acclimated samples is represented largely in PC1, and the contribution of PC1 is much higher than the second principal component. The samples incubated in the dark in the presence of DBMIB lay in a position between the illuminated samples and the dark-incubated samples. Scatter plots shown in Additional file 5: Figure S4B show a similar tendency for samples incubated in the dark in the presence of DBMIB, which show a relatively high correlation with the illuminated samples, while the sample incubated in the dark without the inhibitor and the illuminated samples shows a lower correlation (see Additional file 5: Figure S4B). We conducted a Mulcom test on the samples, which led us to a similar conclusion. DBMIB significantly attenuated the dark-dependency of 1,111 among 1,844 dark-regulated genes (see Additional file 6: Figure S5B). Interestingly, therefore, DBMIB inhibited about 60 % of both reduction in the total mRNA amount and dark-regulated genes. The similar effects of the treatment with DBMIB or KCN under dark conditions suggest that the DBMIB-derived de-repression of the dark-induced transcriptional profiles is primarily because of ATP shortage (because of the inhibition of respiration) in the dark. In other words, the dramatic transcriptional shift upon light-to-dark transition seems to require a certain level of ATP, and the maintenance of ATP availability in the dark appears essential for nocturnal transcription profiles.

While *de novo* dark-induced transcription in a small subset of genes must require sufficient ATP, it remains unclear whether *de novo* transcription is necessary for maintenance of relatively high total transcript levels under conditions of low ATP. To address this question, we used a transcriptional inhibitor, rifampicin, to test the expression profile in the presence of DBMIB. Previously, we had demonstrated that rifampicin treatment in the dark (in the absence of DBMIB) rapidly reduced almost any microarray signals to background levels within four hours, indicating that up-regulation of dark-induced genes and maintenance of residual transcript levels in the dark requires *de novo* transcription, and also revealed short half-lives of most transcripts in the dark [1]. As shown in Fig. 2b, within 60 minutes of light-to-dark transition, three representative dark-repressed transcripts were down-regulated in the dark regardless of the presence or absence of rifampicin, while dark-induced genes failed to be up-regulated in the dark in the presence of rifampicin. By contrast, in the presence of DBMIB, our microarray analysis revealed that addition of rifampicin to dark-acclimated samples did not show such substantial reductions in total mRNA levels (Fig. 3, and see Additional files 5: Figure S4B and 6: Figure S5B). The expression profile of the six representative genes in dark-acclimated, DBMIB-treated cells was also essentially similar in the presence or absence of rifampicin (Fig. 2, and see Additional file 4: Figure S3B).

Based on these observations, we suggest that DBMIB treatment in the dark generally attenuated dark-specific expression profiles for both dark-repressed and -activated genes because of ATP shortage. In other words, these results strongly suggest that not only dark-induced transcription (transcriptional initiation), but also global transcriptional repression (RNA degradation) in the dark, are ATP-requiring active responses to environmental changes. Note that some prokaryotic mRNA degradation processes, such as the unwinding of stable 3′ stem-loops in mRNA, have been reported to require ATP [20].

## Conclusions

**Fig. 4:**
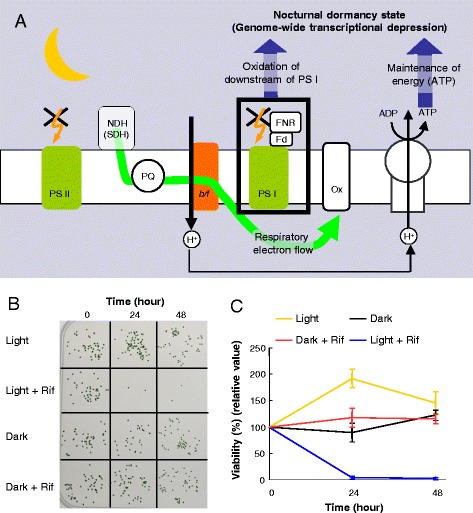
*Synechococcus* actively initiates a nocturnal dormancy-like state. **a** Model of nocturnal dormancy-like state initiation. Initiating nocturnal depression of transcription requires maintenance of ATP content and possibly oxidation of the acceptor side of PS I. PS II, photosystem II; NDH, NAD(P)H dehydrogenase complex; SDH, succinate dehydrogenase; PQ, plastoquinone pool; *b/f*, cytochrome *b6/f* complex; PS I, photosystem I; FNR, ferredoxin; Fd, ferredoxin; Ox, cytochrome-*c* oxidase. **b** *Synechococcus* colonies grown in the light after incubation under different conditions: in the light or the dark in the presence or absence of rifampicin (Rif) for the indicated time. Cells survived in the dark even without *de novo* transcription. **c** The time course of cell survival under each condition quantified from visible colony numbers from independent triplicate experiments. In each experiment, colony numbers were normalized to the average number of control samples at time 0, corresponding to 12 hours in the light. Bars indicate the standard deviation

Our results have shown that *Synechococcus* actively stops transcription and enhances mRNA degradation in the dark via respiration, which requires both the inhibition of photosynthetic electron flow and the maintenance of ATP synthesis via respiration. Considering that ATP content is gradually lowered after longer dark acclimation during the night [5], we assume that dark acclimation triggers the genome-wide transcriptional depression, which is most likely advantageous for suppression of ATP-consuming reactions, such as transcription and translation, in anticipation of the subsequent loss of energy availability during the night. This is a typical feed-forward regulation, which would be important for *Synechococcus* to survive during nocturnal ‘starvation’ time in a relatively inactive fashion, as if they were in a dormant state (Fig. 4a).

Nevertheless, some residual transcription is active in the dark for dark-induced genes, which could also be important for survival in darkness. To address this question, we compared the survival of cells in the dark with or without rifampicin by measuring the number of colony-forming cells. As expected, treatment with the inhibitor dramatically reduces their survival under illuminated conditions (Fig. 4b and c). Surprisingly, however, dark acclimation for at least 48 hours did not affect the survival rates, even in the presence of rifampicin. Thus, dark-acclimated *Synechococcus* cells are resistant to the complete loss of transcription for over 24 hours [1], which supports the view that nocturnal cells are in a dormant-like state. Note that even under such a condition the post-translational oscillation in KaiC phosphorylation is sustained [21], which enables cells to maintain circadian timing at night without *de novo* clock gene expression. Although the circadian regulation is not essential for genome-wide transcriptional depression in the dark, it is able to modulate the magnitude of dark-induced expression of some genes in a time-of-day-dependent manner [2]. Thus, further analysis of the relationship between the possible feed-forward transcriptional depression mechanism and its circadian modulation should be addressed for a more comprehensive understanding of the nocturnal dormant-like strategy in obligate autotrophic organisms.

## Methods

### Strains, culture, and inhibitor treatments

We used wild-type *Synechococcus elongatus* PCC 7942 for all experiments. For all analyses, the initial inoculation of *Synechococcus* cells was at an initial OD_730_ of about 0.075 in BG-11 media [22] and cultured at 30 °C and 40 μmol⋅m^−2^⋅s^−1^. The cells were then acclimated with two LD cycles (except for the samples used in measurements of photosynthetic activity), followed by incubation in the light for 12 hours (cell density with OD_730_ of about 0.20 to 0.35), and then divided and transferred to each condition. To inhibit photosynthesis, we used 2 μM DCMU or 10 μM DBMIB. For controls, we added ethanol, the solvent of the two inhibitors. Note that we confirmed that administration of ethanol in the light for 30 and 60 minutes did not change expression profiles. For the experiments in which respiratory electron flow was inhibited under dark conditions, 1 mM KCN was applied, and a corresponding volume of water was added in all control samples that lack KCN. Rifampin was used at 100 μg/mL. Note that treatment by the inhibitor at this concentration completely inhibited all dark-induced gene expression in the dark (see Additional file 4: Figure S3B).

### Transcriptional analysis

For northern blotting and microarray analyses, we harvested cells and stored them at −80 °C until use. We extracted RNA as described previously [23], subjected it to electrophoresis, blotted it onto nylon membranes, and hybridised it with digoxigenin-labelled DNA probes [2] prepared using DIG DNA Labeling Mix (Roche, Mannheim, Germany) according to the manufacturer’s protocol.

### Microarray analysis

We preformed DNA microarray analyses using Affymetrix GeneChips (Affymetrix, Santa Clara, USA) based on the *Synechococcus* genome as described previously [1, 2]. The data deposited in the National Center for Biotechnology Information Gene Expression Omnibus database (accession no. GSE55637) were divided according to their genomic DNA-normalized values as described in Ito *et al*. [1].

### Data normalization and analysis

For each series of microarray experiments, we further normalized the expression level *x* of gene *g* under *c* condition at *t* hour after transfer to each condition in the nth experiment as:

$$ x\left(g,c,t,n\right)=x\left(g,c,t,n\right)/Y(n), $$

where *x* represents the genomic DNA-normalized expression of gene *g* at *t* hour after transfer to each condition by the procedure described below, *n* ∈ {1, 2, 3, 4}, and used a normalization factor *Y*(*n*) so that the total RNA signal at time 0 (at 12 hours in the light, *l*, at which time each stimulus was added) was deemed to be 1,000, as follows:

$$ Y(n)={\sum}_g\left(x\left(g,l,0,n\right)\right)/1000. $$

Additionally, we normalized each expression datum including northern hybridisation analysis by factor *Z (g*, *n)* so that the expression level of gene *g* at time 0 in each experiment was deemed to be the average of the level at time 0 in all experiments, as follows:

$$ x\left(g,c,t,n\right)=x\left(g,c,t,n\right)/Z\left(g,n\right); $$

$$ Z\left(g,n\right)=x\left(g,l,0,n\right)/\left({\displaystyle {\sum}_n\left(x\left(g,l,0,n\right)\right)/n}\right). $$

The values for the RNA signals given hereafter are these normalized values. For PCA, the correlation matrixes of normalized signals were prepared. We conducted the PCA using an R package [24].

To extract significantly changed transcripts in all 2,515 open reading frames, we used the Mulcom test, a modified version of Dunnett’s multiple comparison test [10]. First, we prepared the matrix including *dx*(*g*, *c*, *t*, *n*), the difference from the control value (at time 0), as follows:

$$ dx\left(g,c,t,n\right)=x\left(g,c,t,n\right)\hbox{--} x\left(g,l,0,n\right). $$

We tested whether there was at least one point where the average of *dx*(*g*, *c*, *t*, *n*) is differentially expressed from the value of continuous illuminated samples, *dx*(*g*, *l*, *t*, *n*) using the Mulcom test. Two parameters in the Mulcom test, *m* and *t*, were determined using the *mulOpt* function in the Bioconductor software package (version 1.2.0), so that the optimal combination of *m* and *t* was chosen to obtain the maximum number of differentially expressed genes satisfying a false discovery rate threshold of <0.003. Additional file 9 describes the parameters and *t* value of the modified Dunnett’s test. Note that the transition of the index for differentially regulated genes is continuous between the regulated and non-regulated genes, and there is no clear cut-off point. Therefore, different thresholds can increase or reduce the number in each group. In the overlapping area of Additional file 6: Figure S5, we counted the number of shared genes if the direction of up- or down-regulation is the same in all groups. For example, *gifA* in Additional file 6: Figure S5A, is not only differentially expressed, but also up-regulated in the dark, light with DCMU, and light with DBMIB, and thus classified in the area shared by any of the three groups in Additional file 6: Figure S5.

### Quantification of intracellular ATP

We extracted ATP from intact cyanobacterial cells according to the method described by Sunamura *et al.* [15]. Briefly, 200 μL of cells in liquid culture were mixed rapidly with 40 μL of chilled 12 % perchloric acid by vortexing, and neutralised with 500 μL of 2 M Tris-acetate (pH 7.7; we confirmed the pH of the mixture to be about 7.5), and then stored the mixture at −80 °C. ATP content was determined using the method described by Rust *et al*. [5]. Thawed extracts (100 μL) were diluted × 1/2.6 in L buffer (25 mM KCl, 50 mM MgSO_4_ and 100 mM HEPES, pH 7.4), and loaded into the wells of a black plastic 96-well plate (Nunc; Thermo Scientific, Waltham, USA). As an internal standard, 0, 5, 10, or 15 pmol of ATP was preloaded. We rapidly added 40 μL of a luciferase mixture (6.25 μg/mL luciferase (firefly recombinant), 250 mM d-luciferin, and 1 mM DTT in Reaction buffer) (Invitrogen; Thermo Scientific, USA) to each sample and then loaded the plate onto a microplate reader (Infinite M200-W; Tecan, Männedorf, Switzerland). Plates were shaken in the dark for 30 seconds at 28 °C before measuring the luminescence signal from each well. We determined the ATP content of each well by linear regression from the four internally standardised wells for each sample.

### P700 absorbance changes

P700 oxidation levels were estimated by the absorbance change of cation radical P700^+^ at 810–830 nm using a dual-wavelength emitter–detector unit (ED-P700DW-E; Walz, Germany) and the emitter–detector–cuvette assembly (ED-101US; Walz, Effeltrich, Germany). We irradiated the samples with actinic light using a fibre illuminator (FL-103/E; Walz, Germany) at 40 μmol⋅m^−2^⋅s^−1^. Before measurement, all samples were dark-adapted for two minutes.

### Measurements of quantum yield of photosystem II

To estimate the effect of the two photosynthetic inhibitors on electron influx to PS II, we measured chlorophyll fluorescence using a pulse-amplitude fluorometer (Water-PAM; Walz, Germany). All samples were dark adapted for two minutes before measurement. Minimal fluorescence (Fo), fluorescence under steady-state conditions (Fs), and the maximum fluorescence of light-acclimated cells (Fm′) were used for calculating parameters, Φ_II_ = (Fm′–Fs)/Fm′ [25, 26]. We irradiated the samples with a saturating 0.8-s pulse to determine Fm′.

### Estimation of cell survival

We estimated cell survival by counting surviving colonies grown under the light after dark and/or inhibitor treatment. Cells that had been acclimated under each condition were washed four times with 1 mL fresh BG-11 liquid media, and then diluted to corresponding OD_730_ of 2 × 10^−5^, and then inoculated onto a 1.5 % BG-11 agar plate, containing 1 mM Na_2_S_2_O followed by incubation at 30 °C under illumination (40 μmol⋅m^−2^⋅s^−1^). After 11 days, we scored the numbers of colonies formed.

## Additional files

Additional file 1: Figure S1. Differential inhibition of photosynthetic electron flows by DCMU and DBMIB. (**A**) The schematic representation of electron flows in cyanobacteria shows linear and cyclic photosynthetic chains and respiratory chains. X indicates the target sites of DCMU and DBMIB. In the case of cyanobacteria, photosynthesis and respiration are intermingled on thylakoid membranes. Fd, ferredoxin; FNR, ferredoxin-NADP (+) reductase; PQ, plastoquinone pool; Ox, cytochrome terminal oxidase. *Solid arrows* schematically indicate the formation of a proton gradient. Linear electron flow needs excitation of PS I and PS II, while cyclic electron flow needs excitation of only PS I. Respiratory electron flow occurs without excitation of PS I and PS II. (**B**) The traces of fluorescence signals recorded by Water-PAM (pulse amplitude modulation) in the presence of inhibitors. The *black arrow* indicates the timing of the saturating irradiation pulse. First, we estimated Φ_II_ (quantum yield of PS II) under light and dark conditions without inhibitors. After the treatment of inhibitors (the timing of adding each stimulus is indicated as *red arrows*), we again estimated Φ_II_ and found it decreased to around zero. Additional file 2: Table S1. Information of the targeted genes used for Figs. 1a, 2b-c and Additional file 3: Figure S2. Additional file 3: Figure S2. Temporal expression profiles of a representative dark repressed or induced gene for eight hours from two independent northern hybridisation experiments. We normalized the data for dark-repressed genes to the maximum value of illuminated samples without inhibitors, while the data for dark-induced genes were normalized to the maximum value of dark-incubated samples. Each plot shows the results of two independent northern blot analyses. As in the other experiments, cells were grown in the light, acclimated to two 12 hour/12 hour light/dark (LD) cycles, and then returned to the light. At 12 hours in the light after the LD cycles, we kept cells under the light, acclimated them to the dark, or treated them with DCMU (0.5 μM) or DBMIB (5 μM). Additional file 4: Figure S3. Expression profiles of representative genes obtained from microarray analysis. Each plot shows the results of independent duplicate experiments. We normalized the data as described in Fig. 1a. (**A**) The effect of inhibition of photosynthesis under illumination. (**B**) The effect of inhibiting ATP synthesis and *de novo* transcriptional initiation in the dark. Additional file 5: Figure S4. Scatterplot comparing induction ratios shows the requirement of photosynthetic inhibition with sustaining the ATP level for transcriptional depression. In the scatterplot analysis, an induction ratio (signal at 30 or 60 minutes divided by that at time 0) in gene expression after transferring each condition plotted as Log_2_ values. (**A**) Dark incubated samples against illuminated with or without each inhibitor. (**B**) Illuminated samples against dark-incubated samples with or without inhibitors. Additional file 6: Figure S5. Diagrams of the number of genes repressed or induced by dark acclimation and/or inhibitor treatment. Data obtained from samples incubated in the dark without inhibitors or in the light in the presence of each inhibitor (**A**), and those from samples in the dark without inhibitors or in the dark in the presence of indicated inhibitor(s) (**B**). Additional file 7: Figure S6. Transition of the ATP level when F1Fo-ATPase activity was blocked by DCCD. Data representation is consistent with that in Fig. 1a. Each plot shows the results from two independent experiments. DCCD (15 μM) was used in these experiments. Additional file 8: Figure S7. P700 oxidation in the presence of the two electron transport inhibitors estimated as Δ*A*_810_. (**A**-**C**) Traces showing P700 oxidation and reduction under each condition. (**D**) Half-decay times of dark reduction of P700 in each condition. We fitted reduction curves from light offset to the time the oxidation level reached the basal line to a single exponential function using a semi-Newtonian method (Microsoft Excel) to estimate the half-decay time. Two-tailed Student *t* tests are used for all comparisons: ***P* <0.01, **P* <0.05. Additional file 9:
**Analysed dataset for extracting genes whose expressions were significantly changed under dark or illuminated condition with or without inhibitors.**

## Abbreviations

DCMU: 3-(3,4-dichlorophenyl)-1,1-dimethylurea
DBMIB: 2,5-dibromo-3-methyl-6-isopropylbenzoquinone
DCCD: *N*,*N*′-dicyclohexylcarbodiimide
Fm′: the maximum fluorescence of light-acclimated cells
Fs: Fluorescence under steady-state conditions
PCA: Principal component analysis
PS I: Photosystem I
PS II: Photosystem II
Φ_II_: Quantum yields of Photosystem II, Fo, Minimal fluorescence
