# Supplementary figures and images for: The initiation of nocturnal dormancy in Synechococcus as an active process
Source: BMC Biol. 2015 Jun 10;13:36. doi: 10.1186/s12915-015-0144-2 (PMC4494158; doi:10.1186/s12915-015-0144-2)

**A**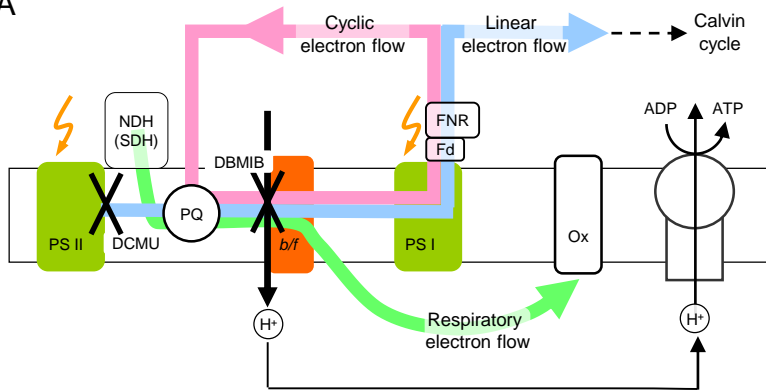**B**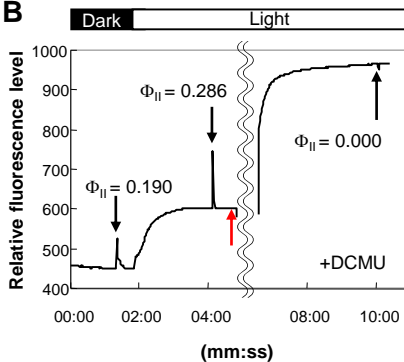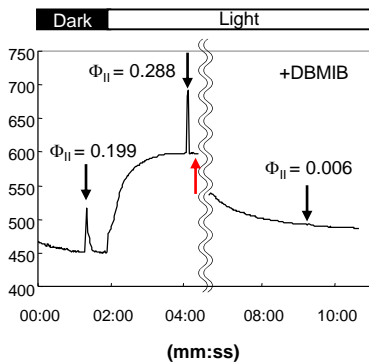

Supplement: Additional file 1: Figure S1. — Differential inhibition of photosynthetic electron flows by DCMU and DBMIB. (A) The schematic representation of electron flows in cyanobacteria shows linear and cyclic photosynthetic chains and respiratory chains. X indicates the target sites of DCMU and DBMIB. In the case of cyanobacteria, photosynthesis and respiration are intermingled on thylakoid membranes. Fd, ferredoxin; FNR, ferredoxin-NADP (+) reductase; PQ, plastoquinone pool; Ox, cytochrome terminal oxidase. Solid arrows schematically indicate the formation of a proton gradient. Linear electron flow needs excitation of PS I and PS II, while cyclic electron flow needs excitation of only PS I. Respiratory electron flow occurs without excitation of PS I and PS II. (B) The traces of fluorescence signals recorded by Water-PAM (pulse amplitude modulation) in the presence of inhibitors. The black arrow indicates the timing of the saturating irradiation pulse. First, we estimated ΦII (quantum yield of PS II) under light and dark conditions without inhibitors. After the treatment of inhibitors (the timing of adding each stimulus is indicated as red arrows), we again estimated ΦII and found it decreased to around zero. [file 12915_2015_144_MOESM1_ESM.pdf]

— Light    — Light + DCMU  
— Dark    — Light + DBMIB

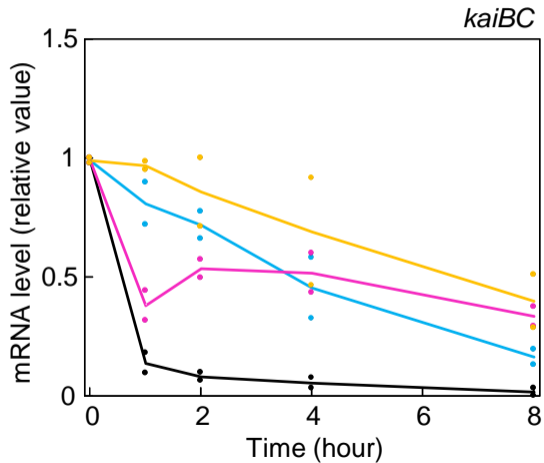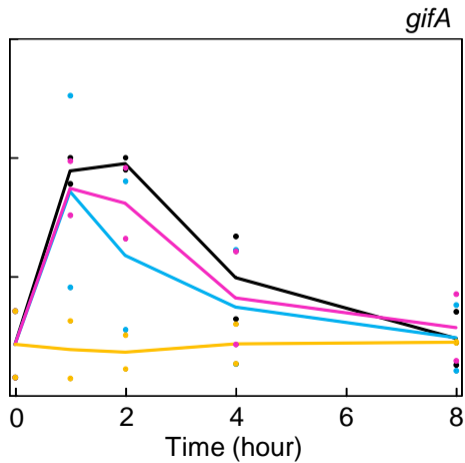

Supplement: Additional file 3: Figure S2. — Temporal expression profiles of a representative dark repressed or induced gene for eight hours from two independent northern hybridisation experiments. We normalized the data for dark-repressed genes to the maximum value of illuminated samples without inhibitors, while the data for dark-induced genes were normalized to the maximum value of dark-incubated samples. Each plot shows the results of two independent northern blot analyses. As in the other experiments, cells were grown in the light, acclimated to two 12 hour/12 hour light/dark (LD) cycles, and then returned to the light. At 12 hours in the light after the LD cycles, we kept cells under the light, acclimated them to the dark, or treated them with DCMU (0.5 μM) or DBMIB (5 μM). [file 12915_2015_144_MOESM3_ESM.pdf]

A

— Light — Light+DCMU  
— Dark — Light+DBMIB

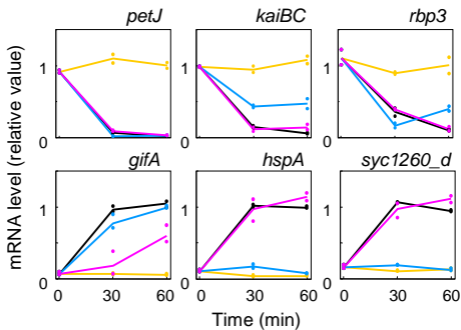

B

— Light — Dark + DBMIB  
— Dark — Dark + DBMIB + Rif

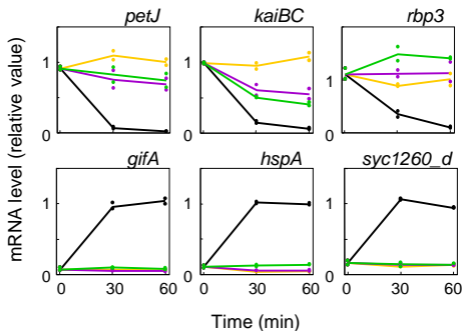

Supplement: Additional file 4: Figure S3. — Expression profiles of representative genes obtained from microarray analysis. Each plot shows the results of independent duplicate experiments. We normalized the data as described in Fig. 1a. (A) The effect of inhibition of photosynthesis under illumination. (B) The effect of inhibiting ATP synthesis and de novo transcriptional initiation in the dark. [file 12915_2015_144_MOESM4_ESM.pdf]

A

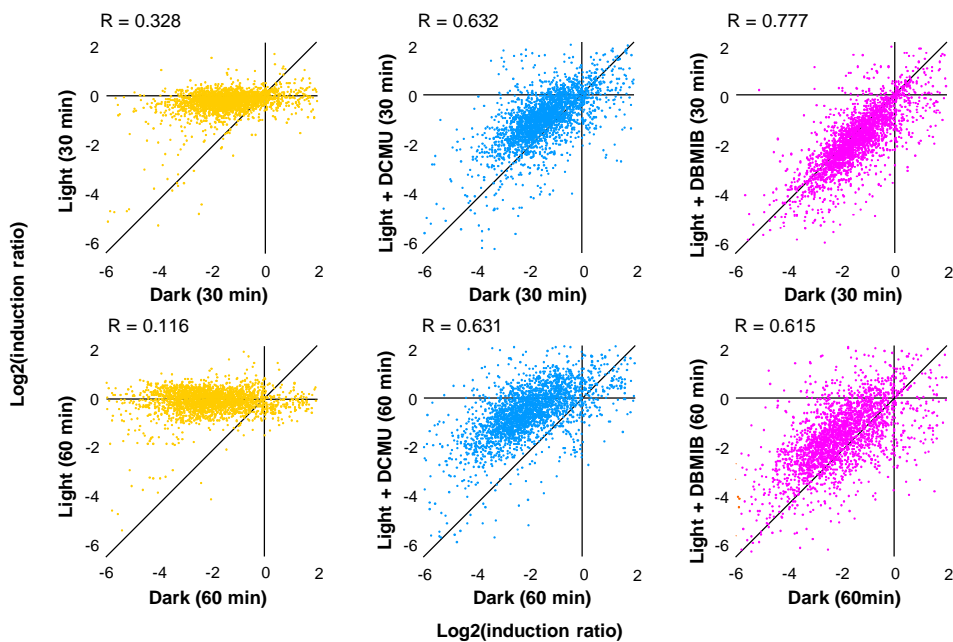

B

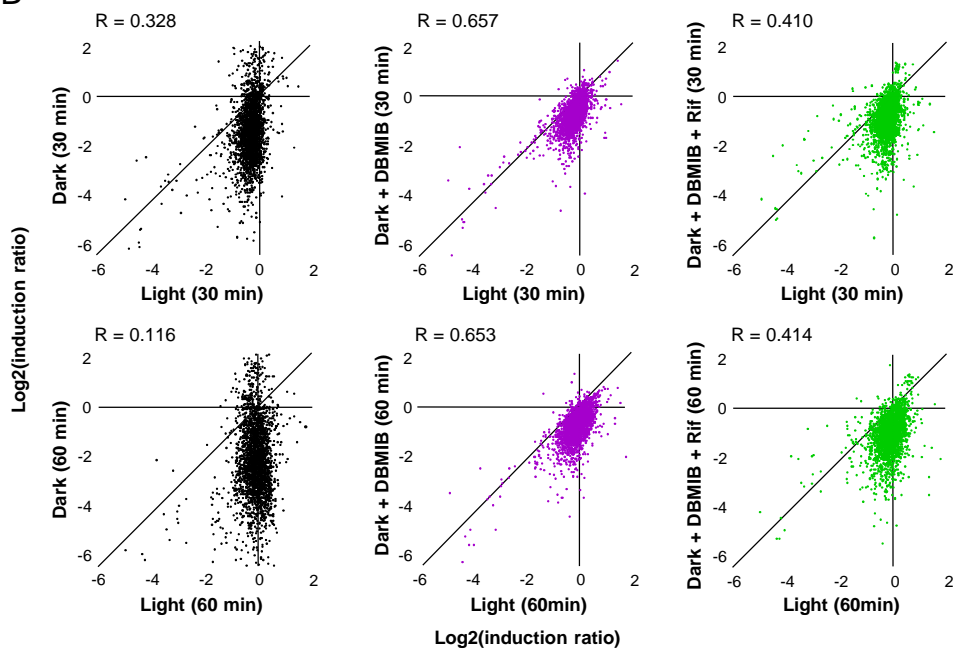

Supplement: Additional file 5: Figure S4. — Scatterplot comparing induction ratios shows the requirement of photosynthetic inhibition with sustaining the ATP level for transcriptional depression. In the scatterplot analysis, an induction ratio (signal at 30 or 60 minutes divided by that at time 0) in gene expression after transferring each condition plotted as Log2 values. (A) Dark incubated samples against illuminated with or without each inhibitor. (B) Illuminated samples against dark-incubated samples with or without inhibitors. [file 12915_2015_144_MOESM5_ESM.pdf]

A

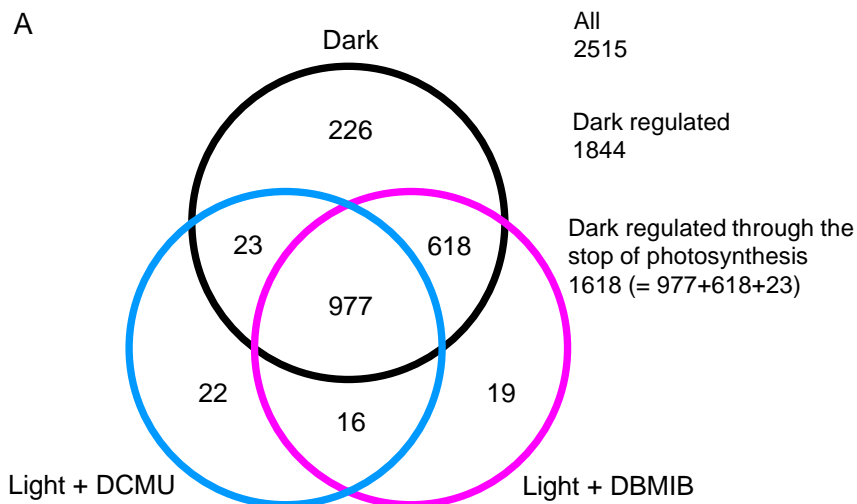

B

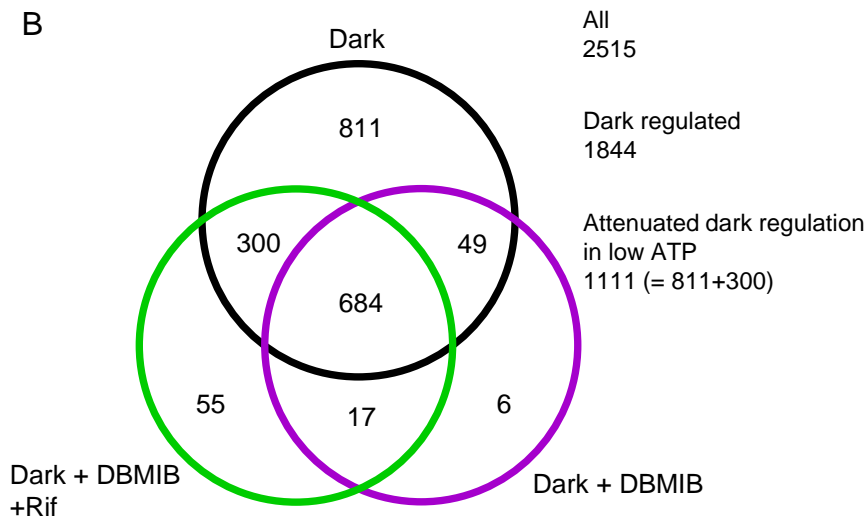

Supplement: Additional file 6: Figure S5. — Diagrams of the number of genes repressed or induced by dark acclimation and/or inhibitor treatment. Data obtained from samples incubated in the dark without inhibitors or in the light in the presence of each inhibitor (A), and those from samples in the dark without inhibitors or in the dark in the presence of indicated inhibitor(s) (B). [file 12915_2015_144_MOESM6_ESM.pdf]

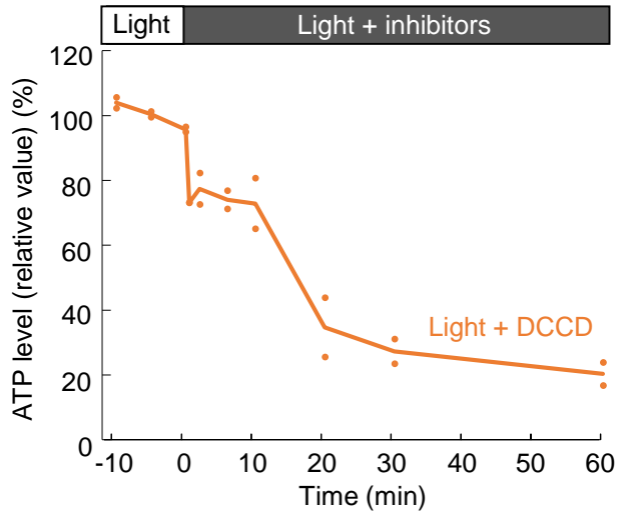

Supplement: Additional file 7: Figure S6. — Transition of the ATP level when F1Fo-ATPase activity was blocked by DCCD. Data representation is consistent with that in Fig. 1a. Each plot shows the results from two independent experiments. DCCD (15 μM) was used in these experiments. [file 12915_2015_144_MOESM7_ESM.pdf]

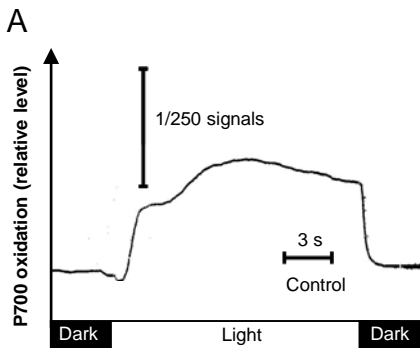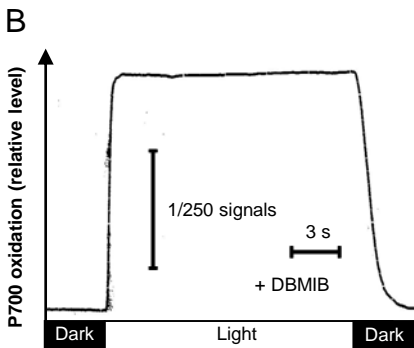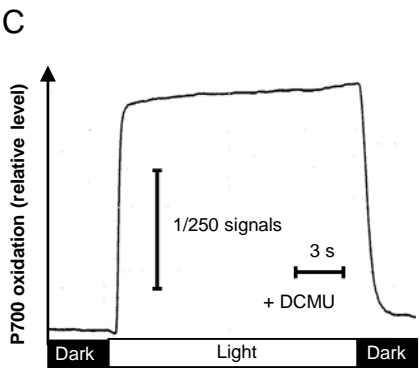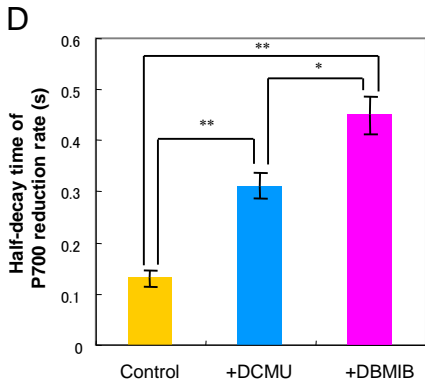

Supplement: Additional file 8: Figure S7. — P700 oxidation in the presence of the two electron transport inhibitors estimated as ΔA 810. (A-C) Traces showing P700 oxidation and reduction under each condition. (D) Half-decay times of dark reduction of P700 in each condition. We fitted reduction curves from light offset to the time the oxidation level reached the basal line to a single exponential function using a semi-Newtonian method (Microsoft Excel) to estimate the half-decay time. Two-tailed Student t tests are used for all comparisons: **P <0.01, *P <0.05. [file 12915_2015_144_MOESM8_ESM.pdf]
