# Supplementary material for: The initiation of nocturnal dormancy in Synechococcus as an active process
Source: BMC Biol. 2015 Jun 10;13:36. doi: 10.1186/s12915-015-0144-2 (PMC4494158; doi:10.1186/s12915-015-0144-2)
Supplement: Additional file 2: Table S1. — Information of the targeted genes used for Figs. 1a, 2b-c and Additional file 3: Figure S2. [file 12915_2015_144_MOESM2_ESM.pdf]

Table S1. Information of the targeted genes used for Figures 1A, Figure 2B-C and Additional file 2: Figure S2.

ID_REF	7942_ID	6301_ID	SYMBOL	DESCRIPTION
syc0089-m_at	Synpcc7942_1630	syc0089_d	*petJ*	cytochrome c553
syc0335-m_at 0336-m_at	Synpcc7942_1217/1216	syc0333_d/0334_d	*kaiB*/*kaiC* operon	circadian clock protein KaiB/KaiC
syc0754-m_at	Synpcc7942_0790	syc0747_d	*rbp3*	RRM-type RNA-binding protein
syc0646-m_at	Synpcc7942_0900	syc0640_d	*gifA*	glutamine synthetase inactivating factor
syc1717-m_at	Synpcc7942_2401	syc1704_d	*hspA*	16.6 kDa small heat shock protein molecular chaperon
syc1272-m_at	Synpcc7942_0253	syc1260_d	-	hypothetical protein
